# New evidence supports *RYR3* as a candidate gene for developmental and epileptic encephalopathy

**DOI:** 10.3389/fneur.2024.1365314

**Published:** 2024-08-16

**Authors:** Jieling Li, Yuexu Ou, Yuanhui Duan, Xiaoming Gan, Hu Liu, Jie Cao

**Affiliations:** ^1^Department of Medical General Ward, Children’s Hospital of Chongqing Medical University, National Clinical Research Center for Child Health and Disorders, Ministry of Education Key Laboratory of Child Development and Disorders, Chongqing, China; ^2^China International Science and Technology Cooperation Base of Child Development and Critical Disorders, Chongqing, China; ^3^Chongqing Key Laboratory of Child Neurodevelopment and Cognitive Disorders, Chongqing, China

**Keywords:** *RYR3*, infantile spasm syndrome, developmental and epileptic encephalopathy, Ca^2+^ release channel, ryanodine receptor 3

## Abstract

**Background:**

The ryanodine receptor 3 (*RYR3*) is involved in skeletal muscle contraction by releasing calcium from the sarcoplasmic reticulum and subsequent T-tubule depolarization. It is also expressed in the brain, and variants in the *RYR3* gene can lead to congenital myopathy type 20 (MIM: #620310).

**Methods:**

We retrospectively analyzed the clinical characteristics and prognosis of a case of West syndrome, developmental and epileptic encephalopathy (DEE) caused by a missense variant in the RYR3 gene. We also reviewed and summarized the literature on epilepsy cases caused by RYR3 gene variants.

**Results:**

A 10-month-old female child with delayed psychomotor development and recurrent spasm-like seizures was diagnosed with infantile spasm syndrome and DEE. Treatment with various antiepileptic drugs resulted in initial improvement but ultimately failed to control the seizures. Whole-exome sequencing revealed a novel heterozygous variant c.10943C > T/p.T3648M in the RYR3 gene, and genome-wide sequencing ruled out other potentially pathogenic variants. Three previous reports have described *RYR3* variants causing DEE, two of which were attributed to *de novo* heterozygous variants, and one was a compound heterozygote.

**Conclusion:**

The present case of DEE caused by a *RYR3* heterozygous variant is consistent with previous rare cases of epilepsy caused by *RYR3* gene variants in terms of pathogenesis and clinical features, but significantly different from congenital myopathy type 20. Our findings provide important evidence for the diagnosis of *RYR3*-related DEE, and we hypothesize that RYR3 gain-of-function variants resulting in “leaky” Ca^2+^ release channels may be a molecular genetic feature leading to DEE rather than myopathy.

## Introduction

When skeletal muscle cells are stimulated, Ca^2+^ is rapidly released from intracellular Ca^2+^ stores in the sarcoplasmic reticulum (SR), a process known as excitation-contraction (E-C) coupling, which leads to a rapid increase in the total Ca^2+^ concentration in the cytoplasm to >0.1 mM. Ca^2+^ release is mainly mediated by ryanodine receptors (RyRs) located on the SR membrane. RyRs form homotetramers and are the largest ion channels discovered to date. There are three types of RyRs in vertebrates: RyR1, mainly expressed in skeletal muscle; RyR2, mainly found in cardiac muscle; and RyR3, which is expressed in trace amounts and predominantly expressed in skeletal muscle and brain (1). All RyRs mediate Ca^2+^-induced Ca^2+^ release (CICR) (2), but in skeletal muscle, CICR is not the main mechanism of physiological Ca^2+^ release (3). It is known that bi-allelic *RYR3* gene variants can cause congenital myopathy (MIM: #620310), consistent with the known function of RYR3 (1, 4). However, the association between RYR3 variants and central nervous system (CNS) disorders is not yet clear.

In this study, we report a case of infantile spasm syndrome and developmental and epileptic encephalopathy (DEE) caused by a heterozygous missense variant in the *RYR3* gene. The molecular genetics and clinical features of this case were consistent with three previously reported cases of *RYR3*-related epilepsy/DEE. This finding not only suggests that *RYR3* variants can cause DEE/epilepsy, but also provides clues for exploring the structure and function of the *RYR3* protein and its role in the CNS through different genotype–phenotype associations.

## Methods

The patient’s parents provided informed consent for the collection of clinical data, which included general information, physical examination, blood and urine tests, electroencephalogram, and MRI. Given the patient’s challenging clinical presentation of intractable infantile spasms and severe developmental and epileptic encephalopathy, genetic testing was performed with approval from medical ethics and the parents’ consent. Trio whole-exome sequencing was performed on the patient and their parents, while single individual whole-genome sequencing was conducted on the patient to detect various types of genetic variations.

A literature search was conducted in PubMed and Google Scholar using keywords “RYR3,” “seizures,” “epilepsy,” “infantile spasm syndrome,” and/or “developmental and epileptic encephalopathy” to identify case reports of RYR3 variants associated with epilepsy and/or DEE.

## Results

A 2-year and 4-month-old female patient presented with delayed psychomotor development and regression. She was the second child of the family’s third pregnancy (G3P2), delivered at 39 weeks by natural childbirth weighing 3.1 kg, with no intrauterine distress, premature rupture of membranes, or perinatal asphyxia. The mother had a normal pregnancy, with a healthy 2+ year-old daughter (G2P1) with no history of seizures or developmental delays. There was a previous unexplained natural miscarriage (G1). Parents were not consanguineous, healthy, and had no family history of seizures or epilepsy.

**Figure 1:**
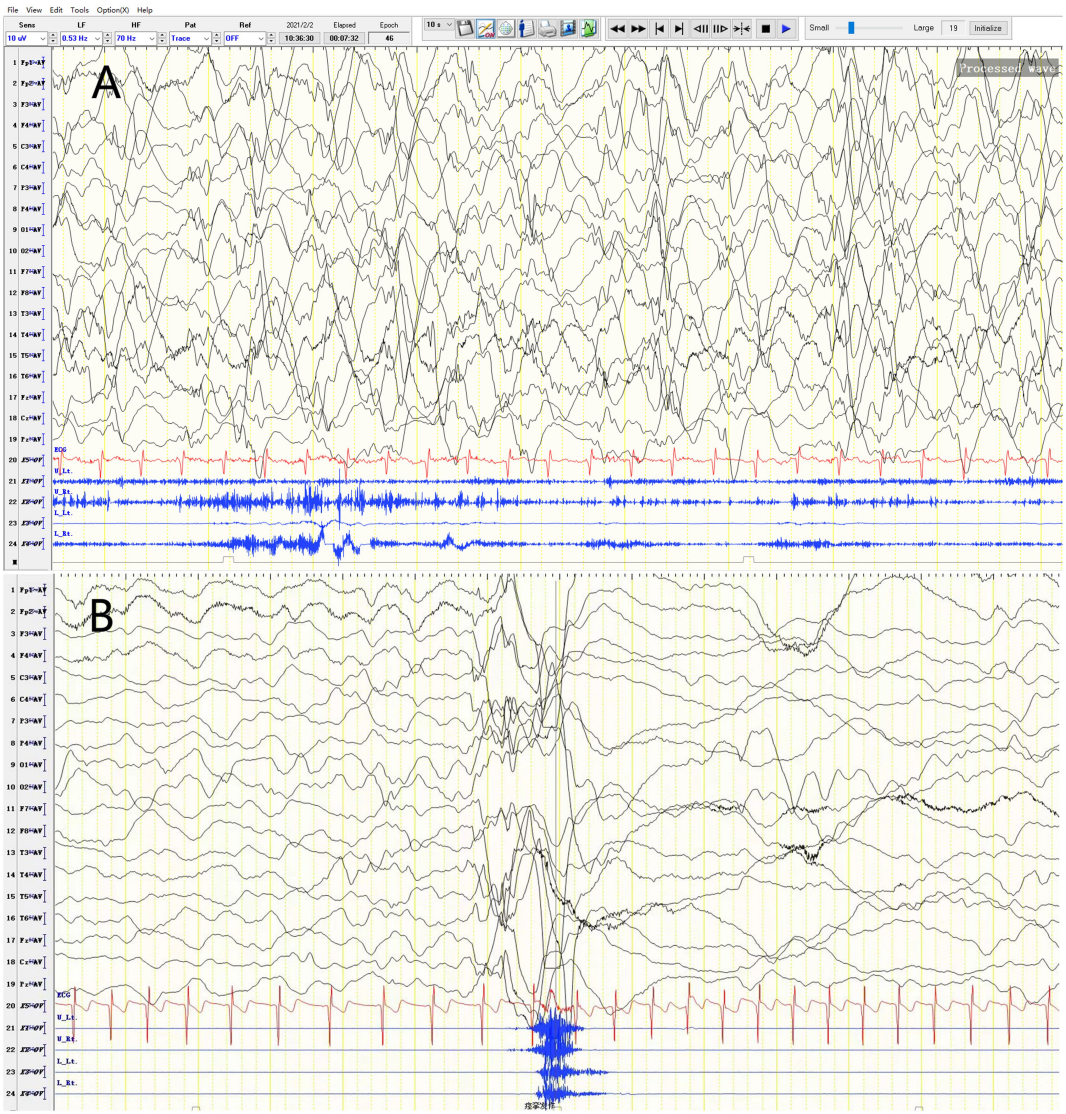
Video EEG of a 2-year-4-month-old female patient. **(A)** Variant-type severe disorganization EEG. **(B)** Ictal period: EEG changes during spasms.

The patient started having unprovoked spasms at 8 months of age, characterized by nodding and bending, mostly occurring before and after sleep. Initially, the spasms occurred once every 4–5 days, and no treatment was given. After one month, the spasms increased in frequency to 3–4 times per day, with each episode lasting 3–4 min and consisting of 30–40 spasms that spontaneously resolved within 10+ seconds. There was no significant delay in psychomotor development. Physical examination revealed a weight of 8.8 kg, head circumference of 43.0 cm, body length of 72 cm, anterior fontanelle size of 0.5*0.5 cm, normal muscle strength and tone in all extremities, and no specific facial features. Blood gas analysis, electrolytes, blood ammonia, lactate, tandem mass spectrometry, and urine organic acid testing were normal. Head MRI revealed no abnormalities. Video electroencephalogram showed multifocal discharges and highly disorganized background activity during the interictal period and spasm-related changes during the ictal period (Figure 1). The clinical diagnosis was infantile spasm syndrome, and treatment with intravenous adrenocorticotropic hormone (ACTH) at 3u/kg/day for 14 days, sodium valproate at 30 mg/kg/day divided q12H, and clonazepam at 0.03 mg/kg/day was initiated. After controlling the spasms, the patient was maintained on long-term treatment with prednisone at 1.5 mg/kg/day, sodium valproate, and clonazepam.

After a proper treatment of prednisone, sodium valproate, and clonazepam for one week, the patient continued to experience frequent spasms, with 3–8 episodes per day and 20–70 spasms per episode. Treatment was adjusted with various antiepileptic drugs, including valproic acid, lamotrigine, topiramate, clobazam, and perampanel. The patient initially improved but relapsed after 1–2 weeks. Currently, the patient is treated with sodium valproate, topiramate, clobazam, and perampanel, along with a ketogenic diet. The patient experiences 3–4 spasm episodes per day, with 10–20 spasms per episode.

The patient’s psychomotor development began to lag and even regress at the age of one. At 2 years and 4 months, the patient can crawl and stand with support but cannot stand or walk independently, can grasp objects but cannot switch hands or point, and can say “ba, ma” unconsciously and recognize strangers.

**Figure 2:**
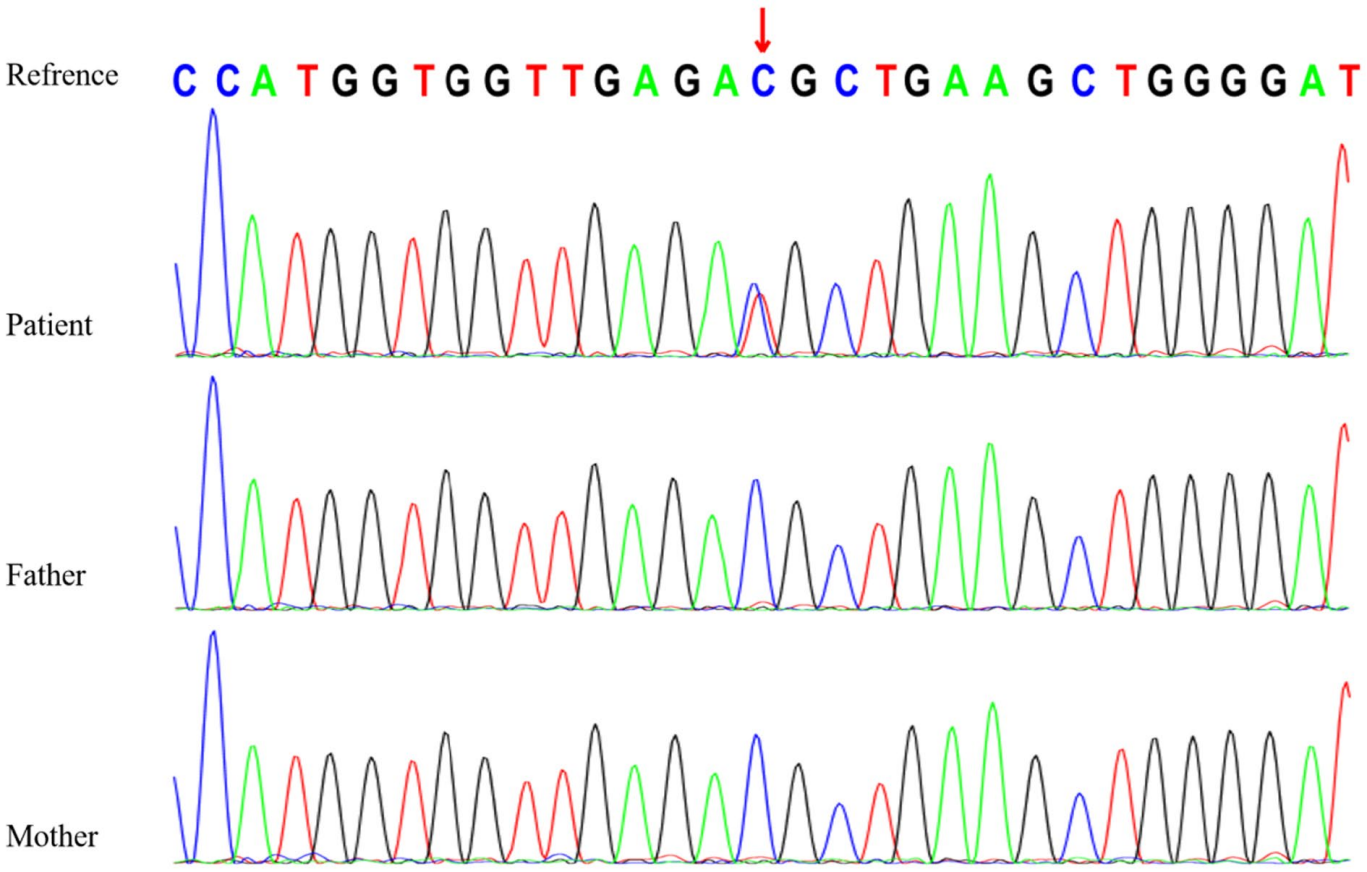
Sanger sequencing of *RYR3* c.10943C>T variant in the family.

Genetic testing revealed a heterozygous variant in the *RYR3* gene (NM_001036:c.10943C > T [p.T3648M]), which is a *de novo* missense variant confirmed by Sanger sequencing (Figure 2). According to the American College of Medical Genetics (ACMG) clinical practice guidelines (5), this variant is likely pathogenic (PS2 + PM1 + PM2 + PP3). However, based on the literature review (Table 1), assuming that *RYR3* variants cause infantile spasm syndrome and/or DEE, this variant is classified as pathogenic according to ACMG criteria (PS2 + PM1 + PM2 + PP3 + PP4). No clinically relevant genomic variants were detected by WGS in the patient.

**Table 1:** Variants in the *RYR3* gene (NM_001036) associated with epileptic encephalopathies in the previously reported cases and this report.

Patient	Nucleic acid change	Protein change	Mutant type	ACMG classification	Disease or phenotype	Author	Year
1	9603_9605delTCA	p.Ile3202del	In-frame deletion	Likely pathogenic	Lennox–Gastaut syndrome	Appenzeller et al. (6)	2014
2	14104G>A	p.Asp4702Asn	Missense	Likely pathogenic	Epileptic encephalopathy	Appenzeller, et al. (6)	2014
3	3716A>G	p.Lys1239Arg	Missense	Uncertain significance	West syndrome	Peng et al. (7)	2018
	4046C>T	p.Thr1349Ile	Missense	Uncertain significance	West syndrome		
4	10943C>T	p.Thr3648Met	Missense	Pathogenic	West syndrome, Developmental and epileptic encephalopathy	Present study	

*De novo* heterozygous variants verified in patient 1 and 2 were classified as “likely pathogenic” by adding evidence PS2.

A search of PubMed and Google Scholar databases yielded two relevant articles (6, 7) reporting three cases of epilepsy/DEE attributed to RYR3 variants (Table 1). In patient 1 and 2 who shared the similar phenotype, Lennox–Gastaut syndrome or DEE, RYR3 variants were confirmed *de novo* that indicates likely pathogenic by adding PS2 evidence, according to the ACMG guideline.

## Discussion

Developmental and epileptic encephalopathies (DEEs) are a group of severe epileptic syndromes characterized by age of onset, seizure types, developmental and clinical course, and EEG findings (8). They are among the most common types of epilepsy in the developmental and epileptic encephalopathy category. Infantile spasm syndrome, characterized by infantile spasms with highly disorganized EEG and developmental delay, is a subtype of DEE. The patient in this case exhibited typical infantile spasm syndrome features and presented with global developmental delay and regression, consistent with a diagnosis of DEE.

In this case, we identified a novel missense variant, RYR3 T3648M. In the absence of a clear association with epilepsy, the ACMG classification of variant T3648M was deemed likely pathogenic. However, when considering previous reports of RYR3 variants in DEE (Table 1), this variant is classified as pathogenic. Additional WGS testing ruled out other variants that could be responsible for the patient’s disease, and sporadic reports suggest that RYR3 variants, particularly heterozygous missense variants, can cause epilepsy and DEE. Therefore, we believe that the patient’s clinical phenotype is attributed to RYR3 T3648M.

The RYR family comprises three distinct genes encoding ryanodine receptors, namely RYR1, RYR2, and RYR3. As calcium release channels in the endoplasmic reticulum, RYRs are widely expressed in all organs, including the central nervous system (9). RYR3 is relatively highly expressed in the brain and is responsible for intracellular calcium release, playing a role in synaptic plasticity. Mice with RYR3 gene knockout exhibit spatial learning impairments (10). Peng Jing et al. (7) proposed RYR3 as a candidate gene for DEE based on a cohort study. The RYR3 variant in this study led to infantile spasms in the patient and subsequent significant developmental delay, even regression. Combined with the video EEG findings of variant-type severe disorganization, the patient was diagnosed with West syndrome and concomitant DEE. The patient’s clinical phenotype differs slightly from that of previously reported RYR3 variant patients (Table 2).

**Table 2:** Clinical characteristics of individuals with RYR3 variants associated with epileptic encephalopathies in the previous literature and present report.

Patient/References	Variant	Gender/Age	Exam at birth	Development prior to epilepsy onset	Sz onset age	Sz type at onset	Other Sz types	Sz outcome	EEG at onset	Course of EEG	Neurological examination/Other features	Development at last follow up	MRI
1 (6)	9603_9605delCAT	M/19y	N	N	8.5 m	Head bobbing	At, T, GTCS	Multiple Sz/d	Multifocal (poly)spikes and slow waves	MFD including slow spikes wave discharge	Normal tonus. Instable gait. Bilateral syndactyly of toes	Severe ID. No speech. Hyperactivity, ASD	Minimal to moderate cerebellar atrophy
2 (6)	14104G>A	M/3y	N	N	6 m	IS	None	Sz free since 11 m	HS	N	N	N	N
3 (7)	3716A>G	F/NA	NA	NA	NA	NA	NA	NA	NA	NA	NA	NA	NA
	4046C>T	F/NA	NA	NA	NA	NA	NA	NA	NA	NA	NA	NA	NA
5	10943C>T	F/2y	N	N	8 m	IS	None	Multiple Sz/d	HS	MFD	N	Severe ID	N

A study found that a West syndrome patient with an *RYR3* gene variant had no seizures after taking calcium antagonists such as sodium valproate, topiramate, and lamotrigine, suggesting that the patient’s seizures may be related to calcium channel defects (9). However, the West syndrome patient in this study did not respond well to calcium antagonists such as sodium valproate, topiramate, and lamotrigine, and still experienced relapses. This suggests that besides calcium channel defects, there may be other unknown factors influencing seizures. Since there are few known RYR3-DEE cases, further studies with more samples are needed in the future.

Lehnart et al. (10) found that the mouse ryanodine receptor 2 (RyR2) R2474S variant can specifically cause spontaneous generalized tonic–clonic seizures without arrhythmias, suggesting that the R2474S variant causing “leakage” of Ca^2+^ release channels in the brain can cause seizures in mice. In the heart, this variant leads to exercise-induced sudden cardiac death. This discovery emphasizes the phenotypic differences of the same RYR variant in different organs. However, the widespread expression of *RYR3* in the human body cannot explain how its variants lead to DEE or myopathy, two completely different clinical phenotypes, and there have been no reports of individuals having both epilepsy and myopathy. Therefore, we believe that different types of RYR3 variants lead to different physiological and pathological changes, which tend to affect the function of different tissues, suggesting a significant genotype–phenotype correlation for RYR3. This different genotype–phenotype correlation for RYR3 may be based on the effect of the variant, such as gain-of-function (GOF) or loss-of-function (LOF), but further basic research is needed to prove this.

In summary, based on the findings of this case and previous case reports, we propose that RYR3 gene variants can lead to DEE, and the RYR3 site of this type of variant has functional differences from the site of variants causing congenital myopathy. In addition, this case demonstrates the application of WGS in clinical diagnosis by ruling out potential other pathogenic genetic factors.

## Data availability statement

The datasets presented in this article are not readily available because of ethical and privacy restrictions. Requests to access the datasets should be directed to the corresponding author.

## Ethics statement

The studies involving humans were approved by Institutional Review Board at Children’s Hospital of Chongqing Medical University. The studies were conducted in accordance with the local legislation and institutional requirements. Written informed consent for participation in this study was provided by the participants’ legal guardians/next of kin. Written informed consent was obtained from the individual(s), and minor(s)’ legal guardian/next of kin, for the publication of any potentially identifiable images or data included in this article.

## Author contributions

JL: Data curation, Formal analysis, Investigation, Methodology, Project administration, Validation, Writing – original draft, Writing – review & editing. YO: Data curation, Writing – original draft. YD: Investigation, Writing – original draft. XG: Resources, Writing – original draft. HL: Data curation, Writing – original draft. JC: Project administration, Supervision, Writing – review & editing, Writing – original draft.
